# STCGAN: a novel cycle-consistent generative adversarial network for spatial transcriptomics cellular deconvolution

**DOI:** 10.1093/bib/bbae670

**Published:** 2024-12-23

**Authors:** Bo Wang, Yahui Long, Yuting Bai, Jiawei Luo, Chee Keong Kwoh

**Affiliations:** College of Computer Science and Electronic Engineering, Hunan University, Changsha, 410083, China; Bioinformatics Institute (BII), Agency for Science, Technology and Research(A^*^STAR), 138671, Singapore; College of Computer Science and Electronic Engineering, Hunan University, Changsha, 410083, China; College of Computer Science and Electronic Engineering, Hunan University, Changsha, 410083, China; College of Computing & Data Science, Nanyang Technological University, 639798, Singapore

**Keywords:** cellular deconvolution, spatial transcriptomics, cycle adversarial network, graph convolutional network

## Abstract

**Motivation:**

Spatial transcriptomics (ST) technologies have revolutionized our ability to map gene expression patterns within native tissue context, providing unprecedented insights into tissue architecture and cellular heterogeneity. However, accurately deconvolving cell-type compositions from ST spots remains challenging due to the sparse and averaged nature of ST data, which is essential for accurately depicting tissue architecture. While numerous computational methods have been developed for cell-type deconvolution and spatial distribution reconstruction, most fail to capture tissue complexity at the single-cell level, thereby limiting their applicability in practical scenarios.

**Results:**

To this end, we propose a novel cycle-consistent generative adversarial network named STCGAN for cellular deconvolution in spatial transcriptomic. STCGAN first employs a cycle-consistent generative adversarial network (CGAN) to pre-train on ST data, ensuring that both the mapping from ST data to latent space and its reverse mapping are consistent, capturing complex spatial gene expression patterns and learning robust latent representations. Based on the learned representation, STCGAN then optimizes a trainable cell-to-spot mapping matrix to integrate scRNA-seq data with ST data, accurately estimating cellular composition within each capture spot and effectively reconstructing the spatial distribution of cells across the tissue. To further enhance deconvolution accuracy, we incorporate spatial-aware regularization that ensures accurate cellular distribution reconstruction within the spatial context. Benchmarking against seven state-of-the-art methods on five simulated and real datasets from various tissues, STCGAN consistently delivers superior cell-type deconvolution performance.

**Availability:**

The code of STCGAN can be downloaded from https://github.com/cs-wangbo/STCGAN and all the mentioned datasets are available on Zenodo at https://zenodo.org/doi/10.5281/zenodo.10799113.

## Introduction

The functions of complex tissues are intricately related to the spatial distribution of different cell types [1–3]. The development of spatial transcriptomics (ST) has revolutionized our ability to map gene expression patterns in native tissue contexts, providing unprecedented insights into cellular heterogeneity and spatial tissue [4, 5]. However, initial ST techniques lacked the resolution and gene coverage of single-cell RNA sequencing (scRNA-seq). For example, the popular 10x Visium platform can capture transcriptomes at scRNA-seq scale; however, it employs 55 $\mu $m spots—substantially exceeding the size of typical cells (5–10 $\mu $m) [6, 7], resulting in each spatial spot representing a mixture of various cell types. Due to the sparse and averaged nature of ST data, performing cellular deconvolution to estimate the proportions of cell types or states within each spatial spot is crucial for analysing complex tissues [8–10].

To address this challenge, several cellular deconvolution methods have emerged, broadly categorized into probabilistic-based, non-negative matrix factorization (NMF)-based, and deep learning-based methods. Probabilistic-based methods leverage probability distributions that align with scRNA-seq gene count distributions and employ likelihood-based inference to estimate cell annotation proportions within capture spots. For instance, Cell2location [11] employs variational Bayesian inference with a negative binomial distribution model for gene expression data, enabling efficient cell type abundance inference, although it requires manual hyperparameter tuning. STdeconvolve [12], based on latent Dirichlet allocation, identifies highly co-expressed genes for each cell type, effectively identifying tissue structures but requiring careful handling of cell type mappings. Similarly, SpatialDecon [13] enhances cell abundance estimation accuracy through log-normal regression and background modeling, albeit at the cost of increased computational time.

NMF-based methods seek a non-negative reference matrix that establishes the correspondence between scRNA-seq and ST expression profiles. For example, SpatialDWLS [14] utilizes damped weighted least squares to precisely determine cell type compositions at specific locations, effectively addressing spatial heterogeneity but potentially exhibiting bias when estimating rare cell type proportions in imbalanced datasets. SPICEMIX [15], a probabilistic latent variable modeling method, enhances cell type inference accuracy in ST data but faces challenges in distinguishing cell type patterns without scRNA-seq reference data. On the other hand, SPOTlight [16] achieves high-precision spatial resolution across tissues using NMF regression and non-negative least squares though it lacks integration of spatial location information in its spatial decomposition.

Deep learning-based methods employ specialized neural network architectures and loss functions to estimate cell annotation proportions. For instance, DSTG [17] utilizes a semi-supervised graph convolutional network to recover cell annotation proportions by learning the linkage graph from shared spaces, with its performance heavily dependent on the quality of the learned linkage graph. Tangram [18] maximizes the spatial correlation between scRNA-seq and ST data by rearranging scRNA-seq expression profiles, demonstrating compatibility with various ST data types despite its computational intensity. Conversely, GraphST [19] integrates graph neural networks with self-supervised contrastive learning to align scRNA-seq and ST data, successfully achieving single-cell spatial resolution and constructing high-resolution cellular atlases.

While these methods have shown promise, they often struggle to achieve single-cell resolution or fully leverage spatial information in ST data. To address these limitations, we propose STCGAN, a novel cycle-consistent generative adversarial network for cellular deconvolution in spatial transcriptomics analysis. First, STCGAN employs a cycle-consistent generative adversarial network (CGAN) to pre-train spatial transcriptomic data. By adhering to the cycle consistency principle, we iteratively feed the reconstructed ST data back into the encoder, ensuring consistency between the mapping from ST data to latent space and its reverse mapping. This enables robust modeling of the data distribution and precise estimation of spatial expression patterns across tissue coordinates. Next, we present a novel cellular deconvolution strategy by learning a trainable cell-to-spot mapping matrix. This strategy integrates scRNA-seq data with corresponding spatial locations by projecting the scRNA-seq data into the ST space, thereby achieving accurate reconstruction of cellular distribution in spatial transcriptomic data. Furthermore, we incorporate spatial-aware regularization into cellular deconvolution to preserve the intrinsic spatial structure during deconvolution, ensuring the reconstruction of consistent cellular distributions in the spatial context. The comprehensive evaluations against seven state-of-the-art methods using five simulated and real ST datasets from various tissues demonstrate that STCGAN consistently outperforms the others in cellular deconvolution.

## Materials and methods

**Figure 1:**
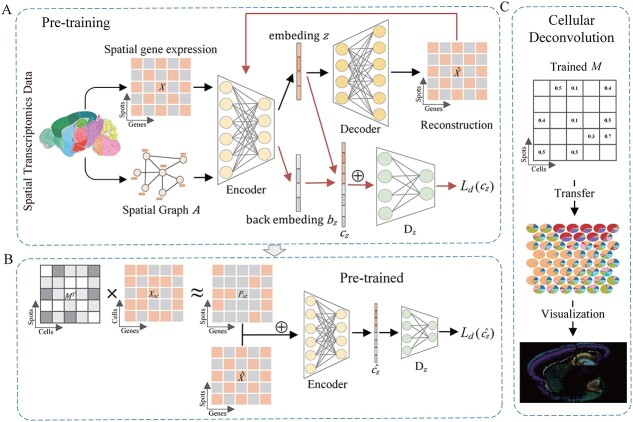
Overview of STCGAN method. (**A**) Pre-training: the VGAE encodes ST data $X$ into embedding $z$ and decodes it to reconstruct expression $\hat{X}$. Next, $\hat{X}$ is re-encoded into back embedding $b_{z}$. Then, the domain discriminator aligns the original embedding $z$ with the back embedding $b_{z}$ into a unified latent space. (**B**) scRNA-seq mapping: a mapping matrix $M$ is trained to transfer scRNA-seq data to the predicted ST matrix $P_{st}$. Subsequently, $P_{st}$ is concatenated with $\hat{X}$ and aligned using the pre-trained model. (**C**) Cellular deconvolution: the trained mapping matrix $M$ is used for cellular deconvolution.

STCGAN comprises two primary steps: pre-training and scRNA-seq mapping. In the pre-training stage, a CGAN is employed to acquire the complex spatial gene expression patterns and learn a robust representation $\hat{X}$ of the underlying data distribution (Fig. 1A). In the scRNA-seq mapping stage, we learn a trainable cell-to-spot matrix to map scRNA-seq data to the predicted ST expression matrix $P_{st}$. Subsequently, $P_{st}$ is concatenated with the reconstructed expression $\hat{X}$ for alignment using the pre-trained network (Fig. 1B). Furthermore, spatial-aware regularization is integrated to maintain tissue structure. Finally, the trained mapping matrix $M$ is then utilized for cellular deconvolution (Fig. 1C).

### Spatial graph construction

To leverage spatial information effectively, we employ the K-nearest neighbors (KNN) method to establish structural relationships among capture spots. Let $A \in \mathbb{R}^{ N_{spot} \times N_{spot}}$ represents the spatial adjacency matrix. If spot $j$ is among the K-nearest neighbors of spot $i$ based on their Euclidean distance, we set $A_{ij}=A_{ji}=1$; otherwise, we assign $0$.

### Variational graph autoencoder

STCGAN utilizes a Variational Graph Autoencoder (VGAE) with Graph Attention Networks (GATs), specifically employing GATv2Conv [20] as the convolutional layer. This architecture enables effective learning of latent features that capture the underlying structure within the ST data. The VGAE takes the adjacency matrix $A$ and the ST expression matrix $X$ as input, undergoing an optimal transformation to yield the reconstructed expression matrix $\hat{X}$ and adjacency matrix $\hat{A}$ as output. The $0$-th layer of the encoder produces a compressed representation:


(1) \begin{align*}& X^{(1)}=GATv2_{(0)}(X, A)\end{align*}

Subsequently, it generates crucial vectors $\mu $ and $\log (\sigma ^{2})$, denoting the mean and variance of the latent space: 


(2) \begin{align*}& \begin{aligned} &\mu=GATv2_{(\mu)}(X^{(1)}, A) \\ &\log \sigma^{2}=GATv2_{(\sigma)}(X^{(1)}, A) \end{aligned}\end{align*}

The latent variable $z$ is then obtained from $z=\mu +\log \sigma ^{2} * \varepsilon $, where $\varepsilon \sim N(0,1)$.

In the decoding stage, VGAE reconstructs the expression matrix and adjacency matrix using dedicated decoders, $D_{X}$ and $D_{A}$, respectively. The decoder $D_{X}$ mirrors the encoder’s structure, while $D_{A}$ is defined by the inner product of latent variables: 


(3) \begin{align*}& \begin{aligned} &\hat{X}=D_{X}(z) = GATv2_{(2)}(GATv2_{(1)}(z, A), A)\\ &\hat{A}=D_{A}(z)=sigm\left(z z^{\top}\right) \end{aligned}\end{align*}

where $sigm\left (\cdot \right )$ is a logistic sigmoid function.

The objective function minimizes the reconstruction loss for both the expression and adjacency matrix and the Kullback–Leibler divergence between the latent feature distribution and the normal distribution: 


(4) \begin{align*}& \begin{aligned} L_{re}=\left\|X-\hat{X}\right\|_{2}^{2} & +\gamma(E_{q(z \mid X, A)}[\log p(A \mid z)]\\ &-K L[q(z \mid X, A) \| p(z)]) \end{aligned}\end{align*}

where $\gamma $ is a tradeoff parameters, $E_{q(z \mid X, A)}[\log p(A \mid z)]$ is the binary cross-entropy function, and $p(\mathrm{z})=\prod _{i} N(0,1)$.

### Cycle-consistent generative adversarial network

Inspired by CycleGAN [21], STCGAN incorporates a cycle-consistent mechanism to enhance its generalization. This ensures data consistency during the transformation between source and target domains, encouraging the preservation of underlying spatial distribution across iterative transformations. Specifically, the encoder first encodes ST data into a latent embedding $z$. The decoder then reconstructs ST expression $\hat{X}$ from $z$, and $\hat{X}$ is re-encoded to generate back-embedding $b_{z}$. This cycle enforces $b_{z}$ to closely match the original $z$.

To address inconsistent embedding feature distributions, we introduce a domain discriminator $D_{z}$, a two-layer multi-layer perceptron (MLP) network that aligns data from different domains ($z$ and $b_{z}$) into a unified latent space. The discrimination loss is articulated as: 


(5) \begin{align*}& L_{d}(c_{z})=-\frac{1}{2N} \sum_{i} \sum_{d=1}^{M} D_{i d} \log \left(p_{i d}\right)\end{align*}

where $$c_{z} = \left [\begin{array}{l}z \\ b_{z}\end{array}\right ]$$ is the concatenated vector containing $z$ and $b_{z}$, $M=2$ represents the number of domains, $D_{id}$ is a sign function indicating if the true domain label of spot $i$ belongs to $z$, assigned $1$ if true, otherwise $0$, and $p_{id}$ denotes the discriminant probability that spot $i$ belongs to domain $z$.

The overall CGAN loss function is: 


(6) \begin{align*}& L_{cyc}= L_{d}(c_{z}) + \alpha L_{re}\end{align*}

where $\alpha $ is a weight parameter.

### Cellular deconvolution

STCGAN introduces an innovative cellular deconvolution strategy that seamlessly integrates scRNA-seq and ST data for accurate cellular deconvolution in spatial transcriptomics analysis. At the core of this strategy lies a trainable mapping matrix $M\in \mathbb{R}^{N_{cell}\times N_{spot}}$, which models the probability of scRNA-seq cells mapping to each capture spot. Specifically, we randomly initialize $M$, where $M_{ij}$ represents the probability of cell $i$ mapping to spot $j$, with $\sum _{i}^{N_{cell}}M_{ij}=1$. This initialization allows the model to start with a broad assumption of cell-to-spot relationships, which are then iteratively refined during training. The predicted spatial gene expression matrix $P_{st}$ is obtained by projecting scRNA-seq expression profiles $X_{sc}$ onto $M$, calculated as: 


(7) \begin{align*}& P_{st}=M^{T}\times X_{sc}\end{align*}

To further refine $P_{st}$ and capture the spatial distribution of ST data, we concatenate $P_{st}$ with the pre-trained output $\hat{X}$. This concatenated input propagates through the pre-trained encoder to yield the embedding $$\hat{c_{z}}=\left [\begin{array}{l}p_{z} \\ b_{z}\end{array}\right ]$$, where $p_{z}$ represents the latent embedding of $P_{st}$. The embedding $\hat{c_{z}}$ is then fed to the domain discriminator. The adversarial training is used to refine the mapping matrix $M$, ensuring it accurately captures spatial distributions while preserving tissue structure. Moreover, STCGAN introduces spatial-aware regularization [22] that leverages spatial neighbor information to optimize the mapping matrix $M$, enhancing the accuracy and robustness of cellular deconvolution. The regularization loss is formulated as: 


(8) \begin{align*}& \mathcal{L}_{reg} =-\sum_{i=1}^{N_{spot}}\left(\sum_{j \in \mathcal{R}_{i}} \log \left(\sigma\left(C_{i j}\right)\right)+\sum_{k \notin \mathcal{R}_{i}} \log \left(1-\sigma\big(C_{i k}\big)\right)\right)\end{align*}

where $C$ denotes the cosine similarity matrix of $p_{z}$, and $\mathcal{R}i$ is the set of spatial neighbors of spot $i$.

By incorporating the regularization, STCGAN encourages the mapping matrix to preserve the spatial tissue structure, further enhancing cellular distribution fidelity. The overall cellular deconvolution loss is: 


(9) \begin{align*}& L_{map} = L_{d}(\hat{c_{z}}) + L_{reg} + \beta \left\|\hat{X}-P_{st}\right\|_{2}^{2}\end{align*}

where $\beta $ is a weight parameter. By optimizing the mapping loss, $M$ is iteratively updated.

Especially, Cellular deconvolution operates independently of scRNA-seq annotations, such as cell types or disease states, enabling flexible projecting of these annotations onto capture spots using the mapping matrix $M$. Formally, let $S \in \mathbb{R}^{N_{cell} \times N_{annot}}$ denote a one-hot annotations matrix, and $N_{annot}$ is the number of annotation labels. The cell annotations probability $P \in \mathbb{R}^{N_{spot} \times N_{annot}}$ across spots can be computed as: 


(10) \begin{align*}& P = M^{T}\times S\end{align*}

To mitigate the influence of low-scoring locations, we empirically retain the top 15% annotation scores for each spot.

### Model training

STCGAN training occurs in sequential to optimize VGAE, CGAN, and cellular deconvolution. First, VGAE is pre-trained for 1000 epochs to effectively capture complex spatial gene expression patterns by minimizing the reconstruction loss $L_{re}$. Then, the CGAN is trained for 500 epochs. During this stage, adversarial loss $L_{cyc}$ is optimized to improve data consistency and refine the reconstructed ST data distribution, with updates to both VGAE and the domain discriminator $D_{z}$. Finally, in the cellular deconvolution stage, the mapping matrix $M$ is fine-tuned over 300 epochs by minimizing the deconvolution loss $L_{map}$, enabling accurate mapping of cell annotations from scRNA-seq data to corresponding spots.

## Results

### Experiment settings

For STCGAN, we set the learning rate to $0.001$ and use a weight decay of 5e-4, optimizing with the Adam optimizer [23]. The selection of highly variable genes (HVGs) differs according to the characteristics of the platform. For instance, the top 3000 HVGs were selected for 10x Visium, the top 5000 HVGs for seqFISH+, and all available genes were used for MERFISH.

To ensure gene consistency, scRNA-seq data is preprocessed using the same methods as the ST data. The weighting parameters $\alpha $, $\beta $ and $\gamma $ are optimized by searching within $\{0.01, 0.1, 1, 10\}$. To find the optimal weighting parameter values, we conducted a parameter analysis using seqFISH+ datasets with 10,000 genes per spot (Fig. S1). The experiments reveal that the optimal performance is achieved with the parameter combination of $\alpha =10$, $\beta =1$, and $\gamma =1$. For performance evaluation, all experiments are repeated 10 times, and root mean square error (RMSE) and Jensen–Shannon Divergence (JSD) are used to evaluate the cellular deconvolution performance on simulated datasets.

### Data preparation and baselines

In this study, we collected five datasets, both simulated and experimentally acquired, paired with their corresponding scRNA-seq datasets: seqFISH+ [24], MERFISH [25], DLPFC [26], Mouse brain anterior and posterior [27], and Human breast cancer [26]. The dataset statistics are summarized in Table S1. Additionally, we selected seven representative methods as baselines: Probabilistic-based methods: Cell2location [11], STdeconvolve [12], and SpatialDecon [13]. NMF-based methods: SpiceMix [15] and SPOTlight [16]. Deep learning-based method: Tangram [18] and GraphST [19]. Notably, SpiceMix and STdeconvolve are reference-free methods that rely solely on spatial locations and spot gene expression profiles from ST data, without requiring external scRNA-seq data for inference. In contrast, the remaining methods require scRNA-seq data from the same tissue as the ST data for accurate estimation.

### Simulations

To evaluate the effectiveness of STCGAN, we performed comparative analyses with seven state-of-the-art methods recommended by [28] on the seqFISH+ [24] and the MERFISH [25, 29] datasets. These methods included Cell2location, GraphST, SpatialDecon, SpiceMix, SPOTlight, STdeconvolve, and Tangram. The seqFISH+ dataset (71 spots and 10,000 genes) exhibited sparse spot distribution but high gene richness, while the MERFISH dataset (3,067 spots and 135 genes) displayed dense spot distribution but limited gene richness. These contrasting attributes provided complementary perspectives, allowing for a comprehensive evaluation of method performance across various scenarios of spot resolution and gene richness.

**Figure 2:**
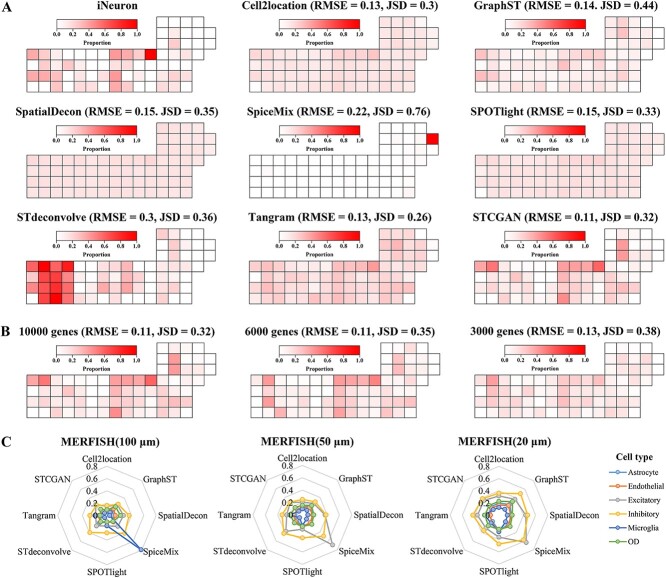
The performance of STCGAN on simulated datasets. (**A**) Visualization of the ground truth and predicted proportions of inhibitory neurons for 8 methods with the seqFISH+ datasets and 10,000 genes per spot. (**B**) The proportions of inhibitory neurons deconvolved by STCGAN for seqFISH+ datasets with three different gene richness (10,000, 6000, and 3000 genes per spot). (**C**) Spider plots showing the RMSE of the deconvolution results for the 8 methods across 6 cell types from the MERFISH at three different spot resolutions (100, 50, and 20 $\mu $m per spot).

In particular, we focus on the distribution of inhibitory neuron cell proportions, which exhibit distinct and localized patterns. Comparisons against ground truth showed that STCGAN consistently outperformed other methods regarding RMSE and JSD scores. Notably, STCGAN achieved RMSE and JSD values of 0.11 and 0.32, respectively, indicating a close match to ground truth patterns (Fig. 2A). Furthermore, we visualized the proportion of inhibitory neuron cells deconvolved (Fig. 2B) by STCGAN across varying gene richness per spot (10,000, 6000, and 3000 genes per spot). The visualizations, accompanied by corresponding RMSE values, demonstrated STCGAN ’s superior performance compared to other state-of-the-art methods, highlighting its robustness across different gene richness. To provide insights into the performance across different cell types, spider plots illustrated RMSE values for all eight methods across various cell types identified in the MERFISH dataset (Fig. 2C). Notably, STCGAN consistently outperformed the other methods across most cell types, except for excitatory neuron and OD cell type, where cell2location exhibited slightly superior performance. Similar results were also observed in the seqFISH+ dataset (Fig. S2). This finding highlights the robustness and effectiveness of STCGAN across diverse gene richness and resolutions.

### STCGAN can predict spatial distributions of cell types in DLPFC

**Figure 3:**
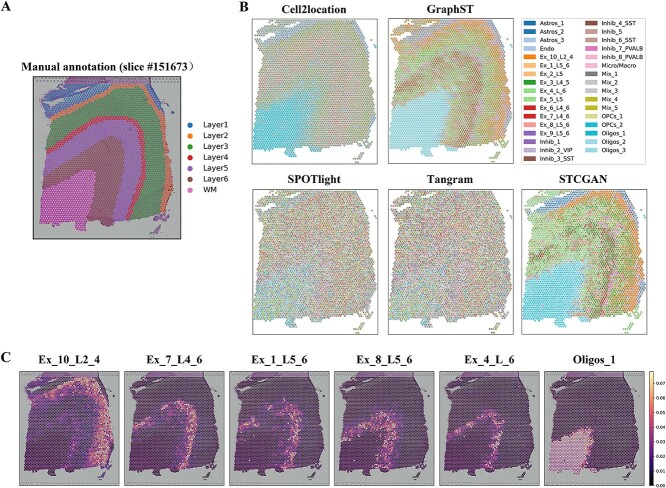
STCGAN predicts spatial distributions of cell types in the slice 151673 of DLPFC. (**A**) Manual annotation of the slice 151673 in DLPFC. (**B**) The spatial scatter pie plots of Cell2location, GraphST, SPOTlight, Tangram, and STCGAN on slice 151673. (**C**) The visualization of the spatial distribution of cell types Ex_10_L2_4, Ex_7_L4_6, Ex_1_L5_6, Ex_8_L5_6, Ex_4_L_6, and Oligos_1 deconvolved by STCGAN on slice 151673.

In this section, we used the DLPFC [26] dataset to validate the cellular deconvolution performance of STCGAN on real-world data. The DLPFC exhibited a clear laminar tissue structure, which can facilitate a better differentiation of performance among deconvolution methods (Fig. 3A).

We evaluated the performance of STCGAN in predicting the spatial distribution of cell types within slice 151673 of the DLPFC dataset. Spatial scatterpie plots demonstrated that STCGAN accurately captured the laminar cortical structure, outperforming other methods (Fig. 3B and S3). In contrast, SpatialDecon, SPOTlight, and Tangram exhibited limitations in distinguishing the laminar tissue structure, likely because their mapping approaches overlook subtle spatial organization in the cortex. While Cell2location was effective in some tissue, it struggled to distinguish layers 2 to 6 clearly due to its modeling approach, which may not capture subtle spatial differences as effectively as STCGAN. Similarly, GraphST blurred the boundaries between layers 2 and 3, as well as between layers 5 and 6, indicating a lack of clear signals between different layers. This may be due to its focus on graph-based connections while overlooking fine-grained layer-specific spatial features. Furthermore, reference-free methods SpiceMix and STdeconvolve failed to effectively distinguish the laminar tissue structure, underscoring the importance of scRNA-seq references in cellular deconvolution. The spatial deconvolution results of STCGAN revealed a unique layer-specific distribution of cell types in DLPFC, showing its advanced capability to reflect cortical physiology and anatomy accurately (Fig. 3C). For instance, STCGAN successfully mapped cell types such as Ex_10_L2_4, Ex_7_L4_6, Ex_1_L5_6, Ex_8_L5_6, and Ex_4_L6 to specific cortical layers, demonstrating the accuracy of the method. Notably, the Oligos_1 cell type was accurately mapped to the white matter (WM) layer, aligning with the high oligodendrocyte concentration in this region. The exceptional performance of STCGAN in the DLPFC slice can be attributed to its novel CGAN framework, which enhances the accuracy of capturing complex tissue structures.

### STCGAN enables horizontal Integration of mouse brain samples

The previous discussion has primarily focused on analysing individual tissue samples, but integrating multiple samples can yield deeper insights [30]. Here, we conducted an integrated analysis of horizontal samples from the mouse brain anterior and posterior [27, 31]. We first aligned their spatial coordinates to construct a joint adjacency graph of the two samples. Subsequently, we utilized it along with their concatenated gene expression for cellular deconvolution.

**Figure 4:**
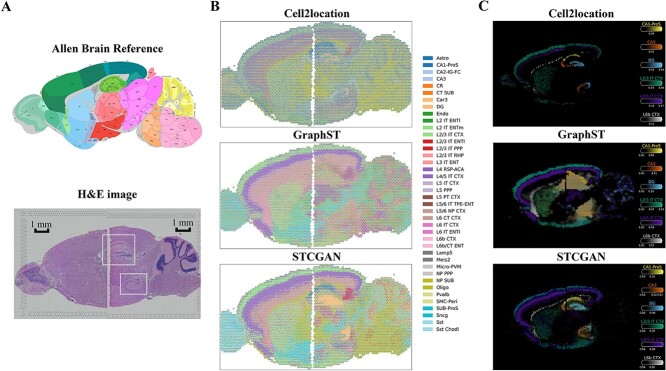
STCGAN horizontally integrates cell types from the mouse brain anterior and posterior. (**A**) Annotated brain section image from Allen Mouse Brain Atlas for reference (top), and H*&*E image of mouse brain anterior and posterior (bottom). (**B**) The spatial scatter pie plots of Cell2location, GraphST, and STCGAN on mouse brain anterior and posterior. (**C**) The estimated cell abundances (color intensity) of hippocampal and cortical layers cell types.

We evaluated the performance of STCGAN across the anterior and posterior mouse brain samples (Fig. 4A, S4–S5). Spatial scatterpie plots indicated that STCGAN effectively captured distinct cell-type patterns, and achieved highly consistent cell-type abundance estimates between adjacent tissue samples. This result suggests the effectiveness of spatial-aware regularization in capturing cellular distribution across different samples. Moreover, the resulting cell-type maps are closely aligned with anatomical locations reported in previous literature (Fig. 4B). In contrast, Cell2location failed to distinguish cortical regions and lacked clear signals across different layers, while GraphST struggled to discern the hippocampus and produced blurred results. SpatialDecon and SPOTlight performed poorly, showing minimal ability to distinguish any tissue structures, while the result of Tangram was heavily affected by noise, further limiting its effectiveness in accurately capturing spatial patterns.

We further analysed the abundances of six cell types (hippocampus: CA1-ProS, CA3, DG, and cortical regions: L2/3 IT CTX, L4/5 IT CTX, L6b CTX) (Fig. 4C). STCGAN accurately depicted the anatomical structures of the cortex and hippocampus, outperforming the other methods. The primary cell types identified by STCGAN at each location were consistent with expectations, affirming its accuracy in spatial mapping. However, Cell2location, SpatialDecon, SpiceMix, and STdeconvolve captured the hippocampus well but encountered a disruption between L4/5 IT CTX and L6b CTX across the two samples. GraphST, SPOTlight, and Tangram performed well in detecting the cortex but struggled with hippocampal structures.

Overall, the integrated analysis using STCGAN showed its superior performance in capturing spatial tissue and accurate cell-type mapping across multiple tissue samples. This provides a deeper understanding of the complex anatomy and heterogeneity of the brain.

### STCGAN can decipher the cellular landscape of human breast cancer

**Figure 5:**
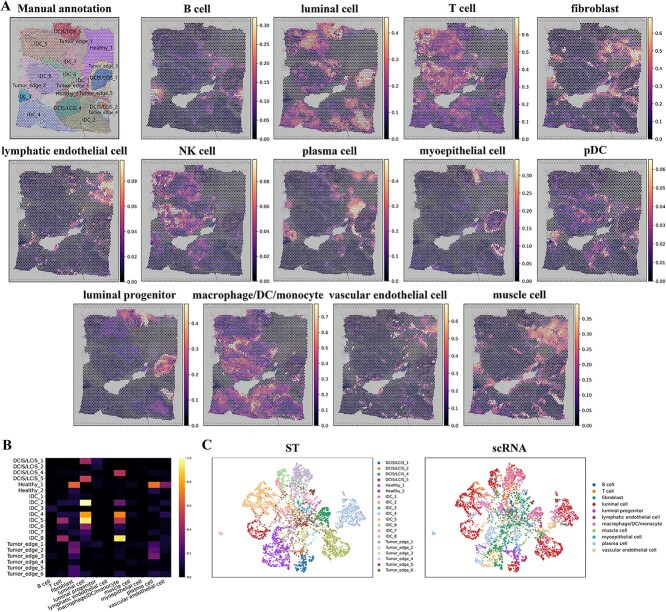
STCGAN deciphers the cellular landscape of human breast cancer. (**A**) Manual annotation and spatial distribution of major cell types mapped by STCGAN, including B cell, luminal cell, T cell, fibroblast, lymphatic endothelial cell, NK cell, plasma cell, myoepithelial cell, pDC, luminal progenitor, macrophage/DC/monocyte, perivascular cell, and vascular endothelial cell. (**B**) Heatmap of the spatial distribution of cell types. (**C**) The visualization of scRNA-seq data and spatial localization of cell types using UMAP, generated from the output cell representations of STCGAN.

In the final analysis, we mapped cell types from the scRNA-Seq breast tissue atlas [32] onto the human breast cancer [26] dataset (Fig. 5A and S6–S12).

We observed that fibroblasts, perivascular cells, lymphatic endothelial cells, and vascular endothelial cells were predominantly mapped to the healthy and tumor edge domains, while myoepithelial cells were mainly localized to the tumor edges. Luminal and luminal progenitor cells were primarily localized within IDC and DCIS/LCIS domains. In comparison, while GraphST, SpiceMix, and STdeconvolve demonstrated the ability to broadly map major cell types, STCGAN’s fine-grained spatial resolution provided a more precise delineation of cell-type heterogeneity and spatial interactions. This distinction highlights the superiority of STCGAN in resolving tissue complexity.

Focusing on immune cells, we found diverse immune subsets in the IDC domains(IDC 5, 6, 7, and 8), including T cells and macrophages/DC/monocytes (myeloid cells). The visualizations in the heatmap and UMAP (Fig. 5B, C) showed correspondence between single cells and their mapped domains. In the DCIS/LCIS domains, only macrophages/DC/monocytes were found in IDC 4 and 5, while no immune cells were mapped to IDC 1 and 2. B cells were mainly localized to IDC 3 and a subset of tumor edge 3, while a small number of plasma cells were found in various tumor edge regions.

The presence of macrophages in tumor tissue has clinical significance, as it is associated with tumor progression by promoting angiogenesis, suppressing immune responses, and facilitating metastasis. High macrophage infiltration frequently correlates with poor patient outcomes, such as reduced overall survival and increased tumor aggressiveness. In contrast, B cells contribute to targeting cancer cells, and their reduced presence in the tumor microenvironment can indicate an impaired immune response against the tumor.

In conclusion, this case study highlights the ability of STCGAN to achieve fine-grained cellular analysis, contributing to a better understanding of cell-type heterogeneity, spatial distribution, and interactions within tissues. STCGAN provides researchers with an advanced tool to explore tissue heterogeneity beyond histological analysis alone, revealing critical insights into cell composition and interactions within tissues.

### Ablation study

To further investigate the working mechanism of STCGAN, we conducted a series of ablation experiments on the seqFISH+ and MERFISH datasets. Specifically, we systematically removed the cycle-consistent constraint, domain discriminator, and spatial-aware regularization to evaluate their contributions to model performance.

- STCGAN-w/o-$L_{cyc}$: it omits the cycle-consistent generative adversarial network during the pre-training stage but a simple variational graph autoencoder.
- STCGAN-w/o-$L_{d}$: it does not utilize the trained domain discriminator in the cell-type deconvolution stage, i.e. $L_{d}$ is excluded from the $L_{map}$ loss.
- STCGAN-w/o-$L_{reg}$: it lacks spatial-aware regularization, so $L_{reg}$ is not included in the overall loss function.

As shown in Table S2, STCGAN consistently outperforms its variants. Specifically, STCGAN demonstrates excellent performance in terms of RMSE score compared to STCGAN-w/o-$L_{cyc}$. The cycle-consistent constraint effectively models the spatial expression estimation of spatial transcriptomic data, improving the accuracy of cell-type deconvolution. Additionally, we observe that STCGAN-w/o-$L_{d}$ has lower RMSE scores but exhibits significant RMSE score variations under different spot resolutions and gene richness. This indicates that the domain discriminator positively contributes to mapping the underlying data distribution, and its removal could lead to unstable performance. Finally, STCGAN-w/o-$L_{reg}$ yields mediocre results across various datasets. The spatial-aware regularization effectively preserves the tissue structure in spatial transcriptomic data, thus improving the accuracy and robustness of cell-type deconvolution. Without spatial-aware regularization, STCGAN faces challenges in effectively integrating spatial structure and gene expression.

Overall, the ablation experiments underscore the importance of integrating the cycle-consistent constraint, domain discriminator, and spatial-aware regularization into STCGAN. The integration of these components enables better cell-type deconvolution, enhancing the model’s performance and stability.

## Discussion and conclusion

In this paper, we propose STCGAN, a cellular deconvolution method based on a cycle-consistent generative adversarial network. We first employ a cycle-consistent adversarial network to capture the complex spatial gene expression patterns, ensuring that the model can accurately depict the spatial structure. Next, we introduce a novel cellular deconvolution strategy that learns a trainable cell-to-spot mapping matrix, effectively transferring cell annotation information from scRNA-seq data to capture spatial spots by projecting the scRNA-seq data into the ST space. Furthermore, we incorporate spatial-aware regularization to enhance the accuracy and robustness of cellular deconvolution.

We conduct experiments on multiple simulated and real ST datasets. The results demonstrate that STCGAN outperforms state-of-the-art methods in cellular deconvolution in spatial transcriptomics analysis. Experiments on simulated datasets show the robustness and effectiveness of STCGAN across diverse gene richness and resolutions. The results of experiments conducted on the DLPFC dataset demonstrate that STCGAN can accurately capture the laminar tissue of the cortex. This can be attributed to STCGAN’s ability to explore subtle spatial tissues through the CGAN framework, providing a more detailed understanding of tissue heterogeneity. In horizontal integration experiments, it is observed that STCGAN captures diverse cellular patterns and ensures seamless cell-type abundance estimates across adjacent tissue samples. This is attributed to the spatial-aware regularization in STCGAN, which facilitates the capture of cellular distributions across diverse samples, yielding more accurate cellular deconvolution. Finally, a case study on a human breast cancer dataset demonstrates that STCGAN enables fine-grained cellular analysis, revealing cell-type heterogeneity, understanding the cell type distribution and interactions within tissues, and offering valuable insights for cancer research and treatment.

Despite the successes achieved by STCGAN in cellular deconvolution, it relies on predefined cellular reference information for deconvolution. This dependency limits STCGAN’s applicability and scope when facing unknown or unrepresented cell annotations in reference datasets. In future work, we aim to address this limitation by leveraging cross-dataset training and transfer learning.

**Key Points**

- In this study, we propose STCGAN, a cycle-consistent generative adversarial network for spatial transcriptomics cellular deconvolution.
- STCGAN employs a cycle-consistent adversarial network to capture the complex spatial gene expression patterns and align scRNA-seq data with the ST space through a trainable cell-to-spot mapping matrix, accurately estimating the cellular composition in each capture spot.
- We evaluated STCGAN on 5 simulated and real datasets from different tissues. Experimental results demonstrate that STCGAN outperforms seven state-of-the-art methods in cellular deconvolution performance, thereby proving its superiority in the field of ST analysis.

## Supplementary Material

Supplementary_materials_of_STCGAN_bbae670

## Data Availability

For the seqFISH+ dataset, both the ST data and the scRNA-seq data are obtained from (https://github.com/CaiGroup/seqFISH-PLUS). The MERFISH dataset is obtained from (https://datadryad.org/stash/dataset/doi:10.5061/dryad.8t8s248/), and the scRNA-seq data is obtained from (https://github.com/rdong08/spatialDWLS_dataset/tree/main/datasets). The DLPFC dataset is obtained from (http://research.libd.org/spatialLIBD/), and the scRNA-seq data is obtained from (https://www.ncbi.nlm.nih.gov/geo/query/acc.cgi?acc=GSE144136). The mouse brain tissue dataset is collected from (https://mouse.brain-map.org/static/atlas), and the scRNA-seq reference is acquired from (https://portal.brain-map.org/atlases-and-data/rnaseq/mouse-whole-cortex-and-hippocampus-10x). The human breast cancer is collected from (https://www.10xgenomics.com/resources/datasets/human-breast-cancer-block-a-section-1-1-standard-1-1-0), and the scRNA-seq data is obtained from (https://www.immunesinglecell.org/).
