# Correction to: STCGAN: a novel cycle-consistent generative adversarial network for spatial transcriptomics cellular deconvolution

**DOI:** 10.1093/bib/bbaf064

**Published:** 2025-02-04

This is a correction to: Bo Wang, Yahui Long, Yuting Bai, Jiawei Luo, Chee Keong Kwoh, STCGAN: a novel cycle-consistent generative adversarial network for spatial transcriptomics cellular deconvolution, *Briefings in Bioinformatics*, Volume 26, Issue 1, January 2025, bbae670, https://doi.org/10.1093/bib/bbae670

In the originally published version of the manuscript, in section Variational graph autoencoder, there was duplication in the last sentence of the first paragraph. This should read only: ``The 0-th layer of the encoder produces a compressed representation:[...]'' intead of: ``The 0-th layer of the encoder produces a compressed representation: the 0-th *GATv2*_(0)_ layer of the encoder produces a compressed representation:[...]''

The correction has been made in the article.

