# Supplementary material for: STCGAN: a novel cycle-consistent generative adversarial network for spatial transcriptomics cellular deconvolution
Source: Brief Bioinform. 2024 Dec 23;26(1):bbae670. doi: 10.1093/bib/bbae670 (PMC11666287; doi:10.1093/bib/bbae670)
Supplement: Supplementary_materials_of_STCGAN_bbae670 [file supplementary_materials_of_stcgan_bbae670.docx]

**Supplementary materials of STCGAN**

**Supplementary Figure**


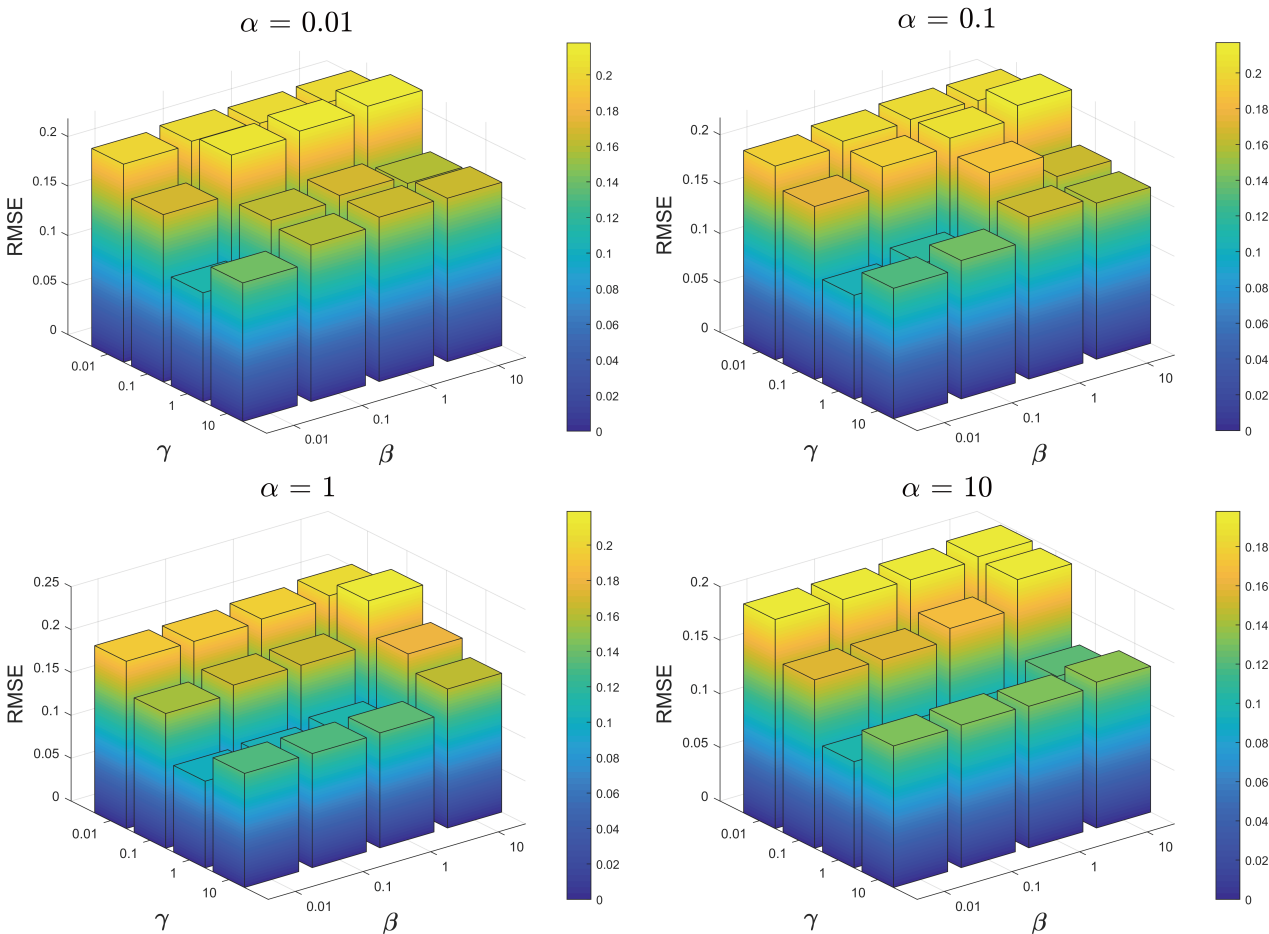


**Fig S1.** The sensitivity analysis of parameters α, β, and γ with the seqFISH+ datasets (10,000 genes per spot).

**
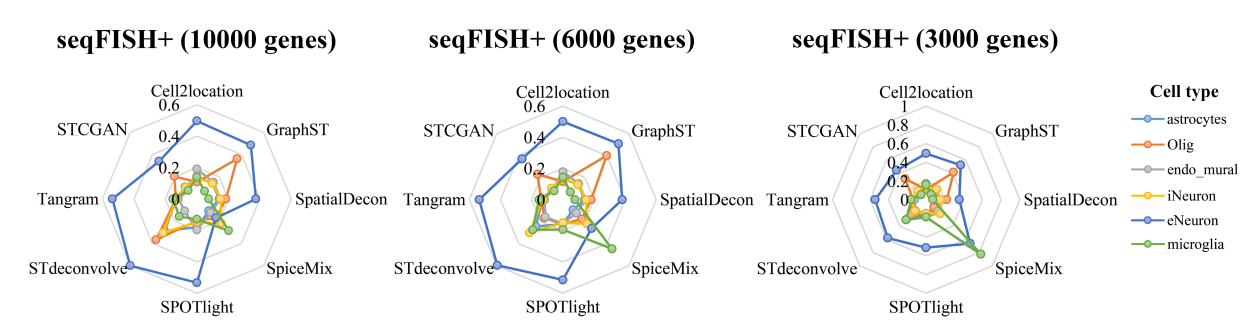
**

**Fig S2.** The spider plots show the RMSE of the deconvolution results for the 8 methods among 6 cell types from the seqFISH+(10000, 6000, and 3000 genes per spot).

**
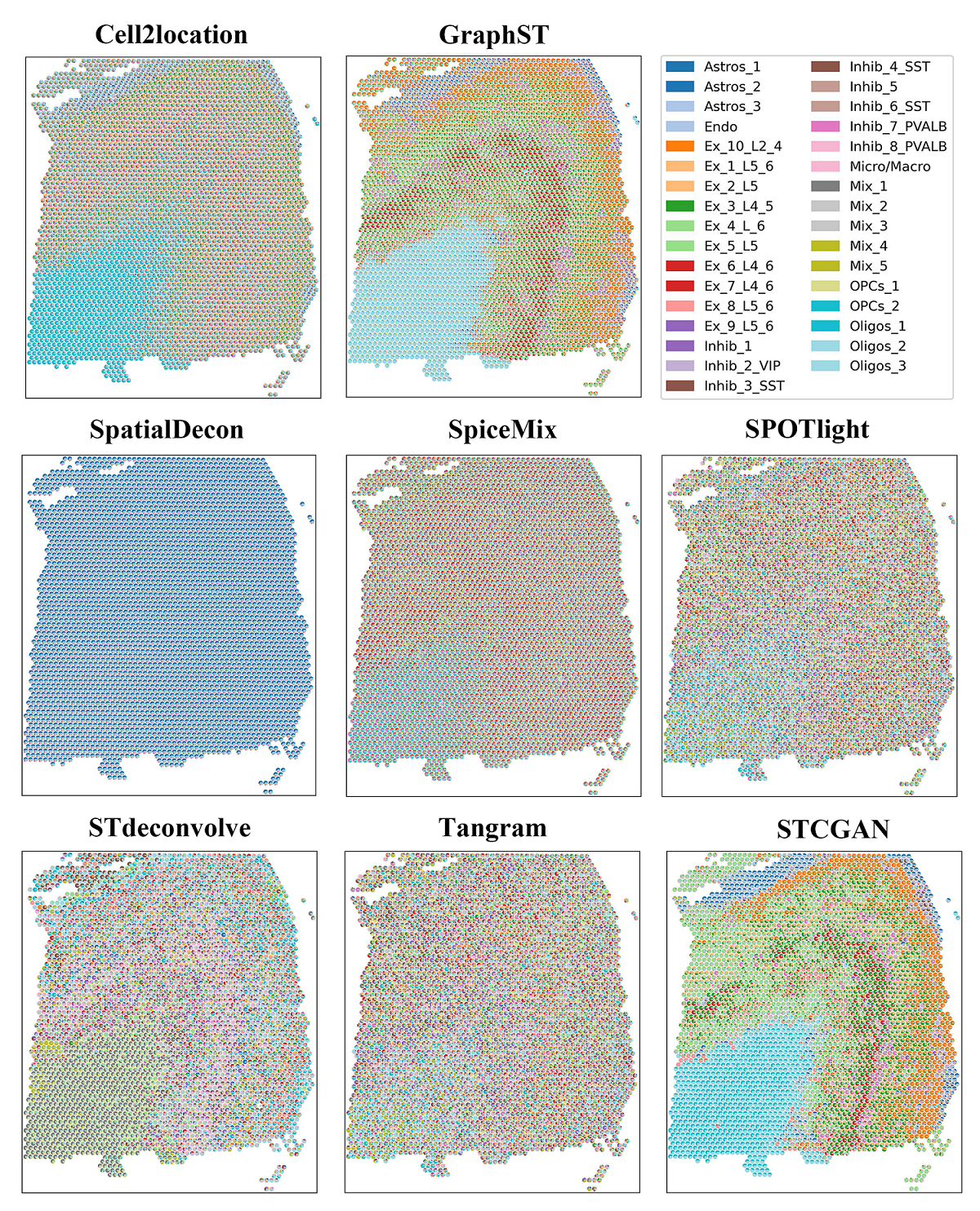
**

**Fig S3.** The spatial scatter pie plots of Cell2location, GraphST, SpatialDecon, SpiceMix, SPOTlight, STdeconvolve, Tangram, and STCGAN on slice 151673.

**
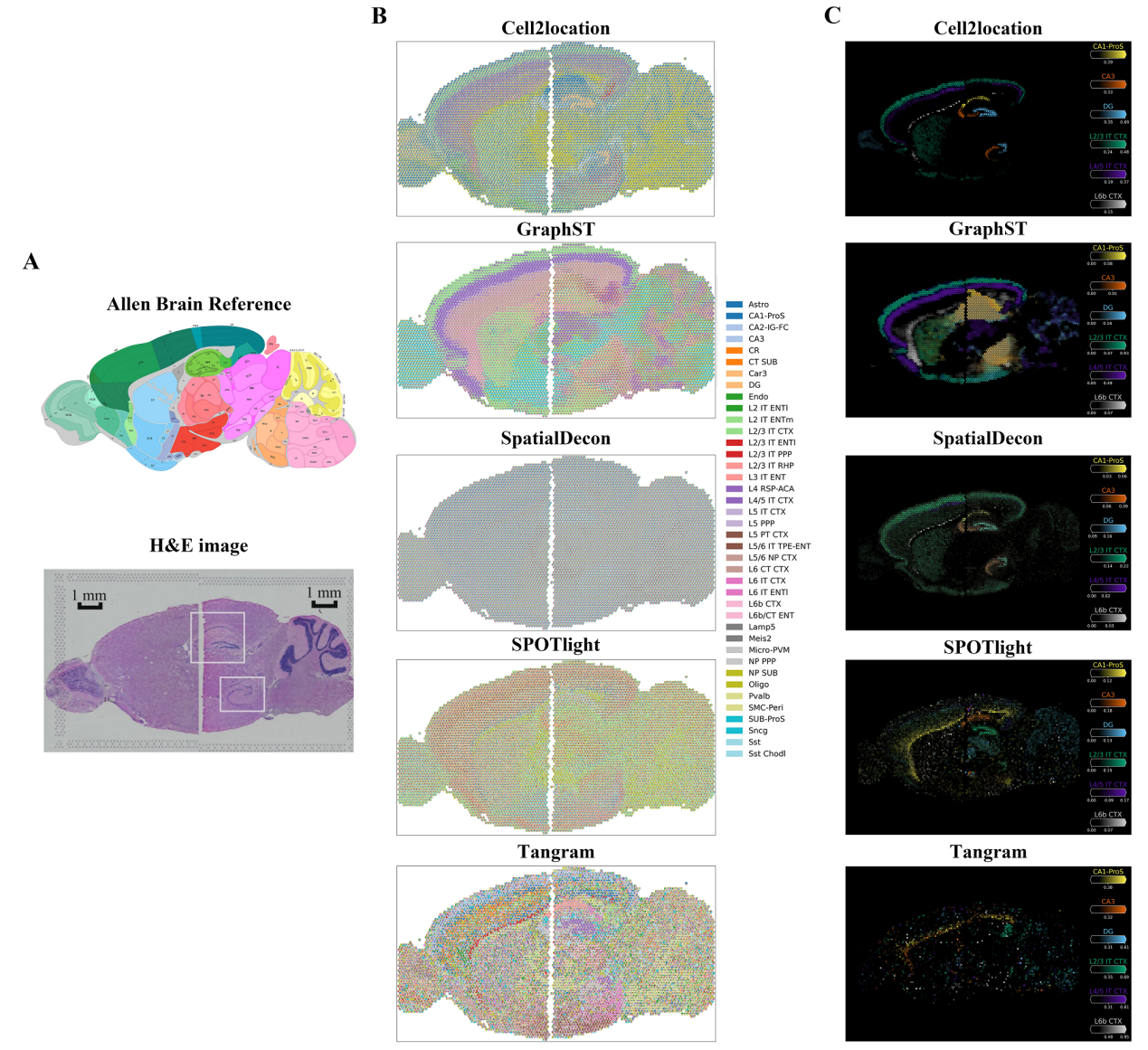
**

**Fig S4.** Horizontal integration of mouse brain anterior and posterior cell types using scRNA-seq reference-based methods. (A) Annotated brain section image from Allen Mouse Brain Atlas for reference (top), and H&E image of mouse brain anterior and posterior (bottom). (B) The spatial scatter pie plots of Cell2location, GraphST, SpatialDecon, SPOTlight, and Tangram on mouse brain anterior and posterior. (C) The estimated cell abundances (color intensity) of hippocampal and cortical layers cell types.

**
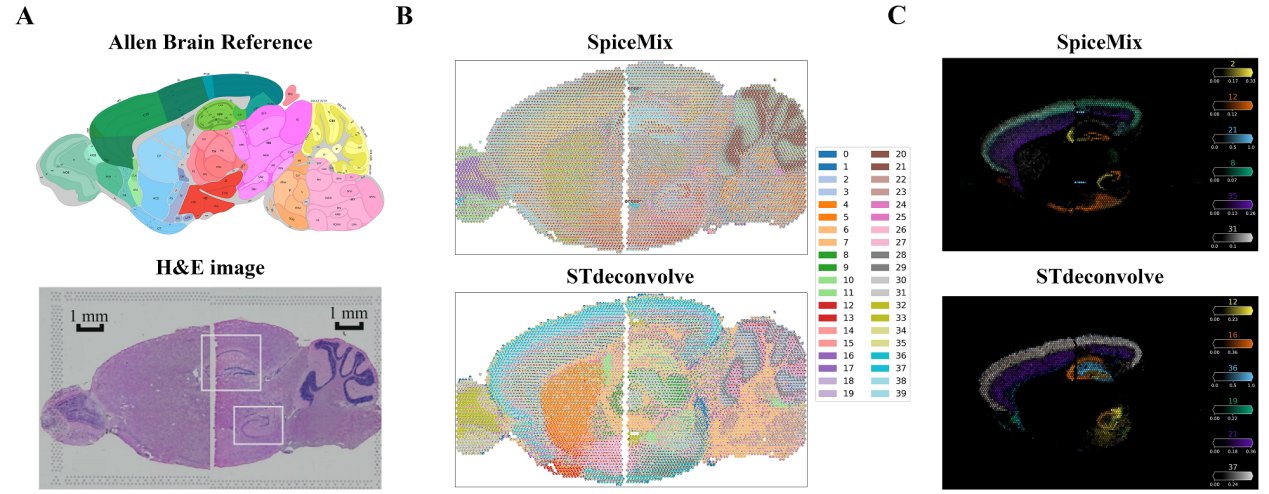
**

**Fig S5.** Horizontal integration of mouse brain anterior and posterior cell types using scRNA-seq reference-free methods. (A) Annotated brain section image from Allen Mouse Brain Atlas for reference (top), and H&E image of mouse brain anterior and posterior (bottom). (B) The spatial scatter pie plots of SpiceMix and STdeconvolve on mouse brain anterior and posterior. (C) The estimated cell abundances (color intensity) of hippocampal and cortical layers cell types.

**
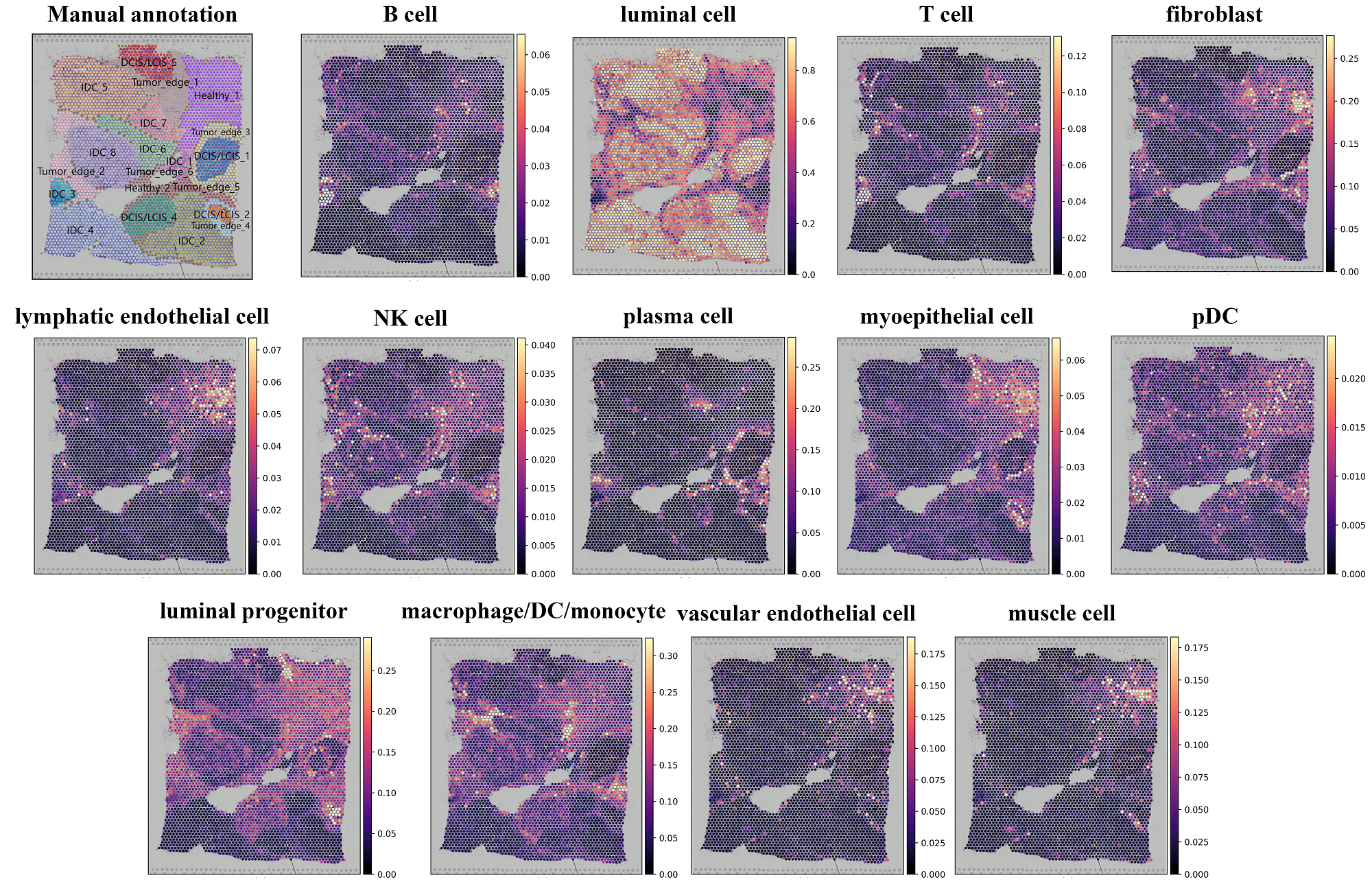
**

**Fig S6.** Manual annotation and spatial distribution of major cell types mapped by Cell2location.

**
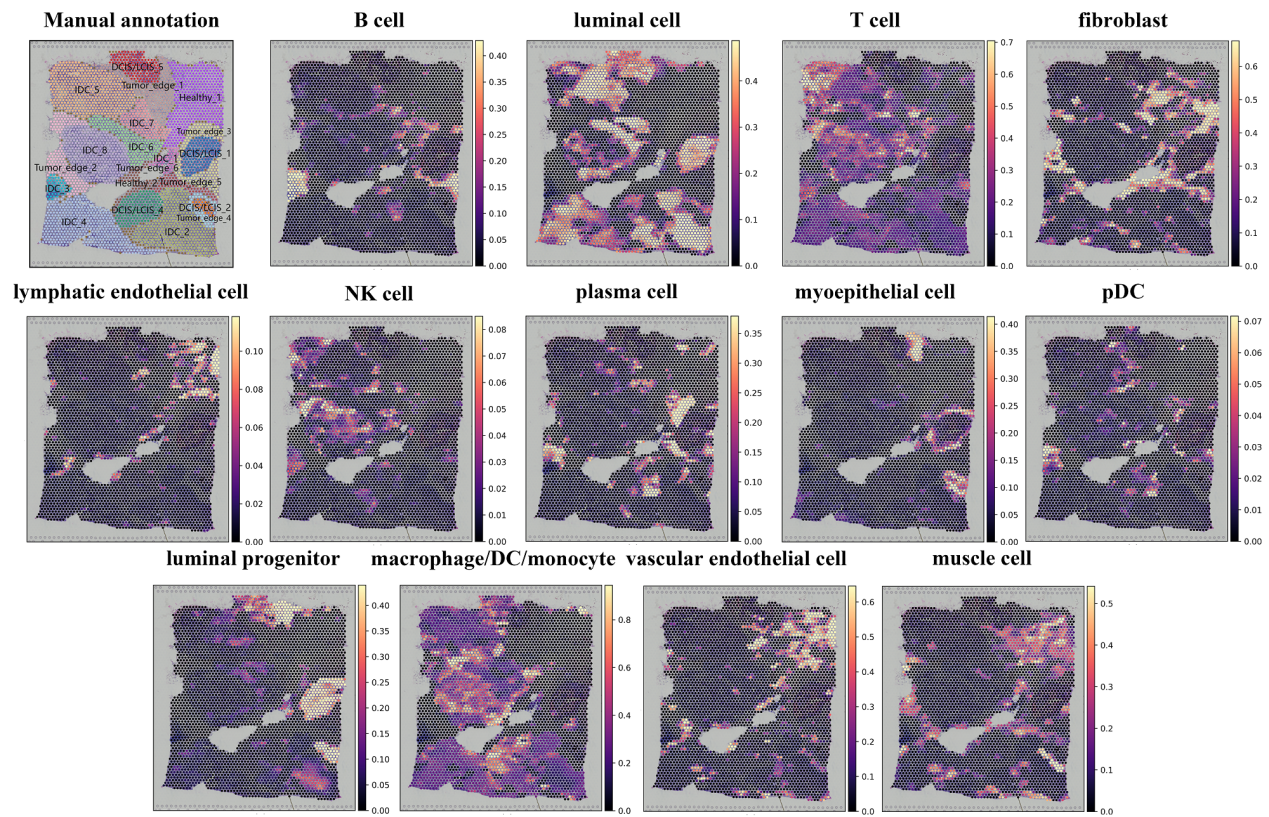
**

**Fig S7.** Manual annotation and spatial distribution of major cell types mapped by GraphST.

**
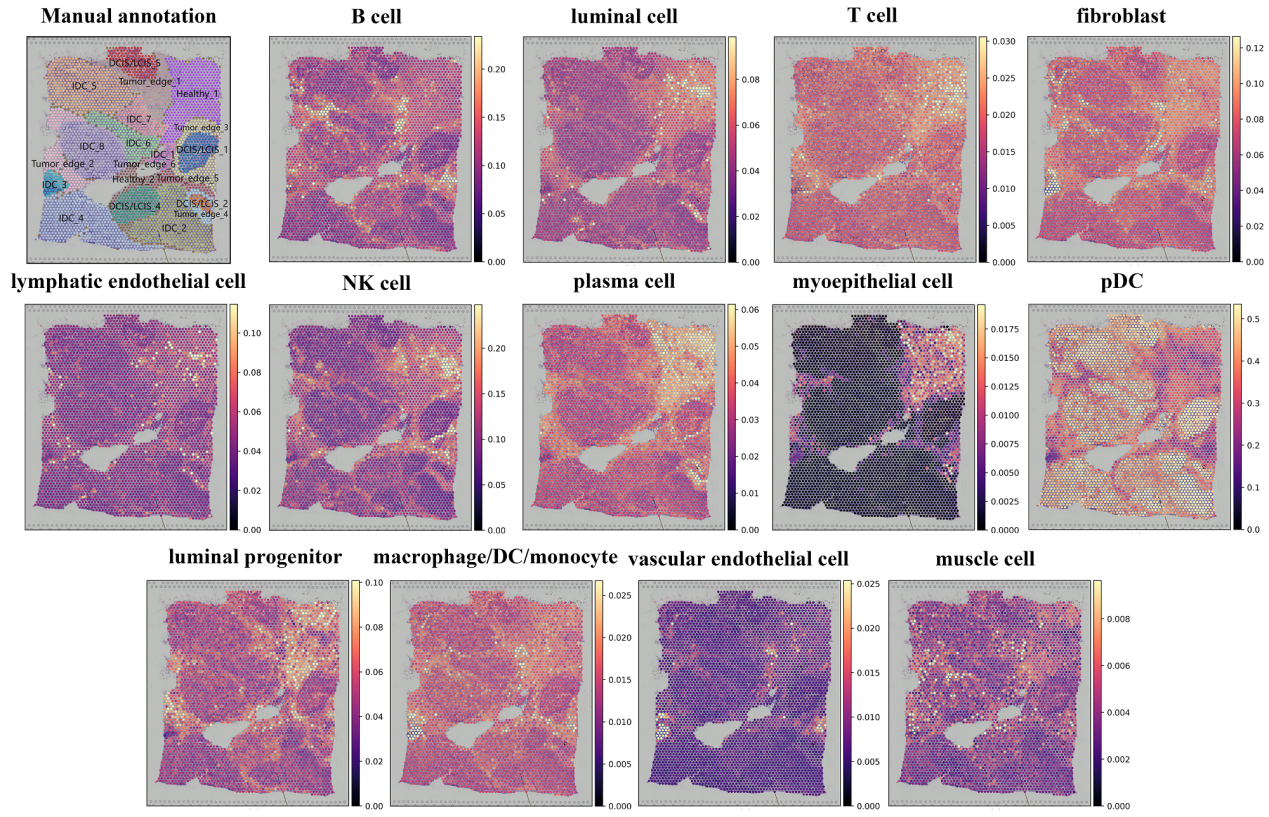
**

**Fig S8.** Manual annotation and spatial distribution of major cell types mapped by SpatialDecon.

**
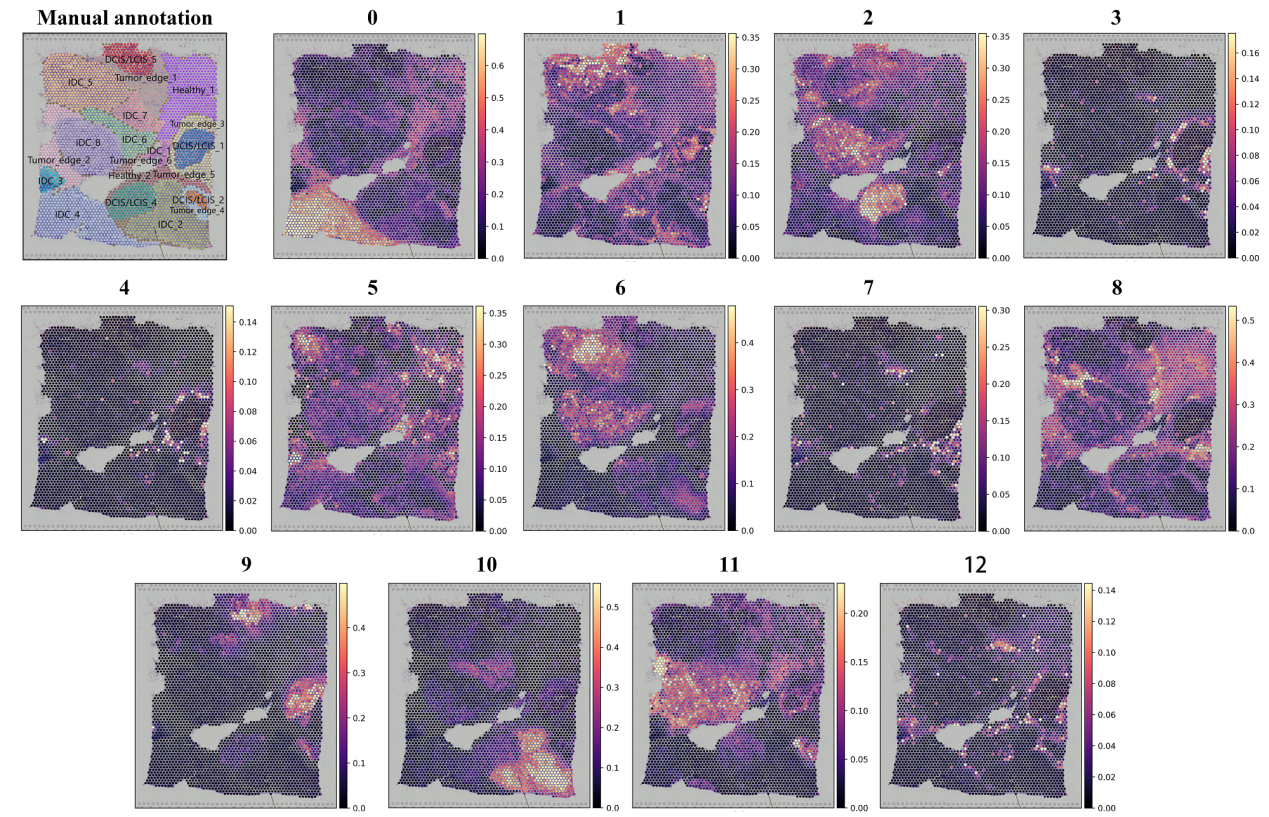
**

**Fig S9.** Manual annotation and spatial distribution of major cell types mapped by SpiceMix.

**
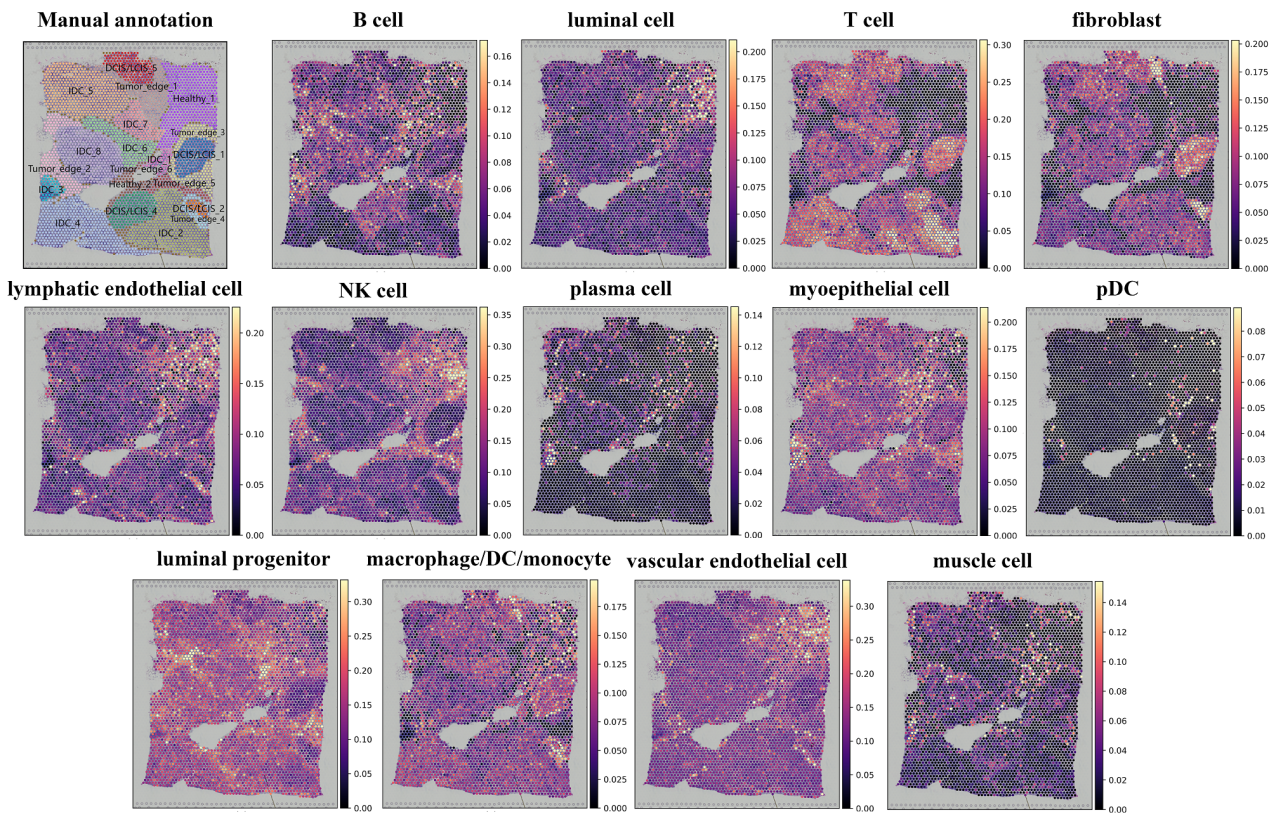
**

**Fig S10.** Manual annotation and spatial distribution of major cell types mapped by SPOTlight.

**
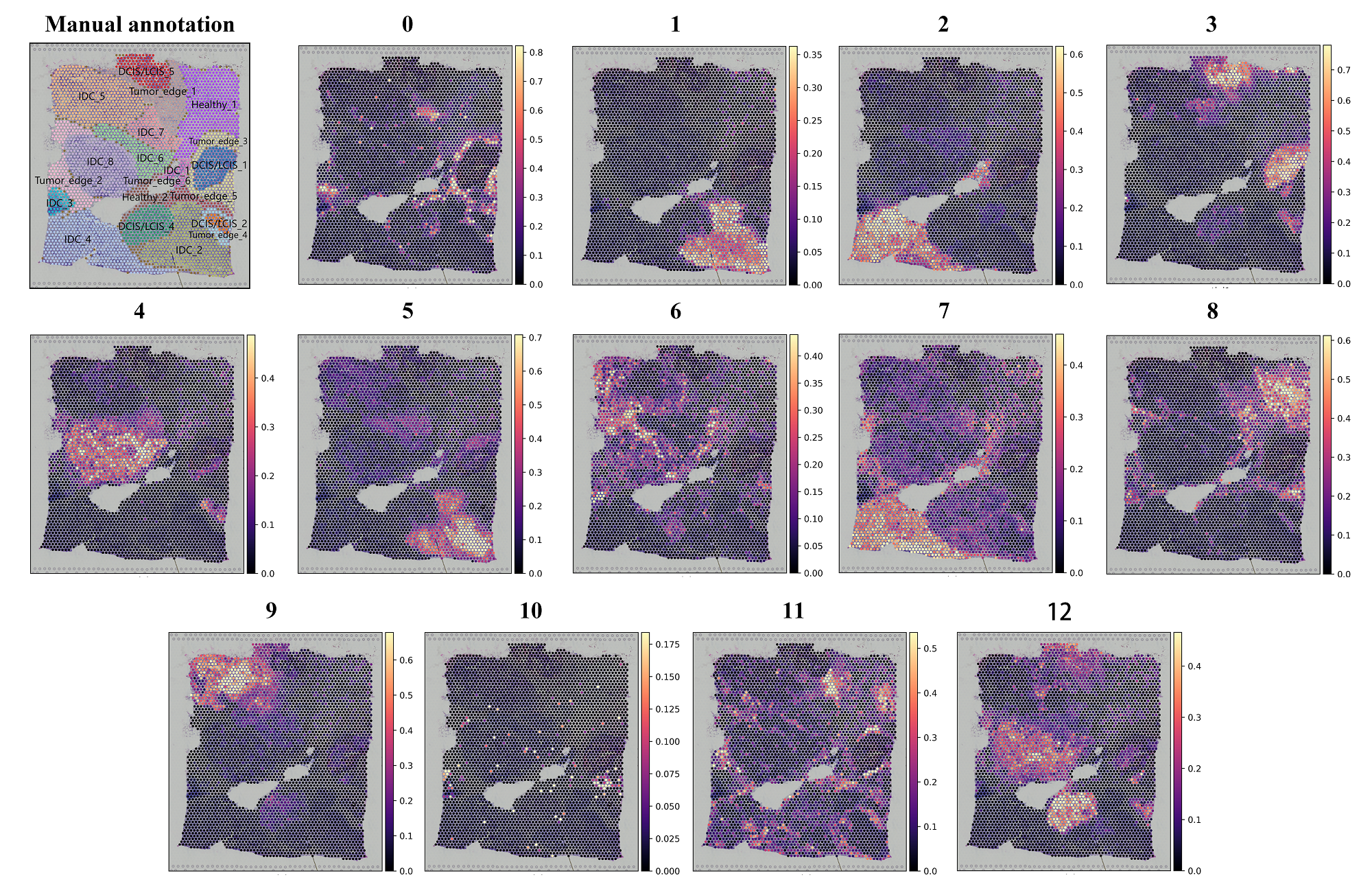
**

**Fig S11.** Manual annotation and spatial distribution of major cell types mapped by STdeconvolve.

**
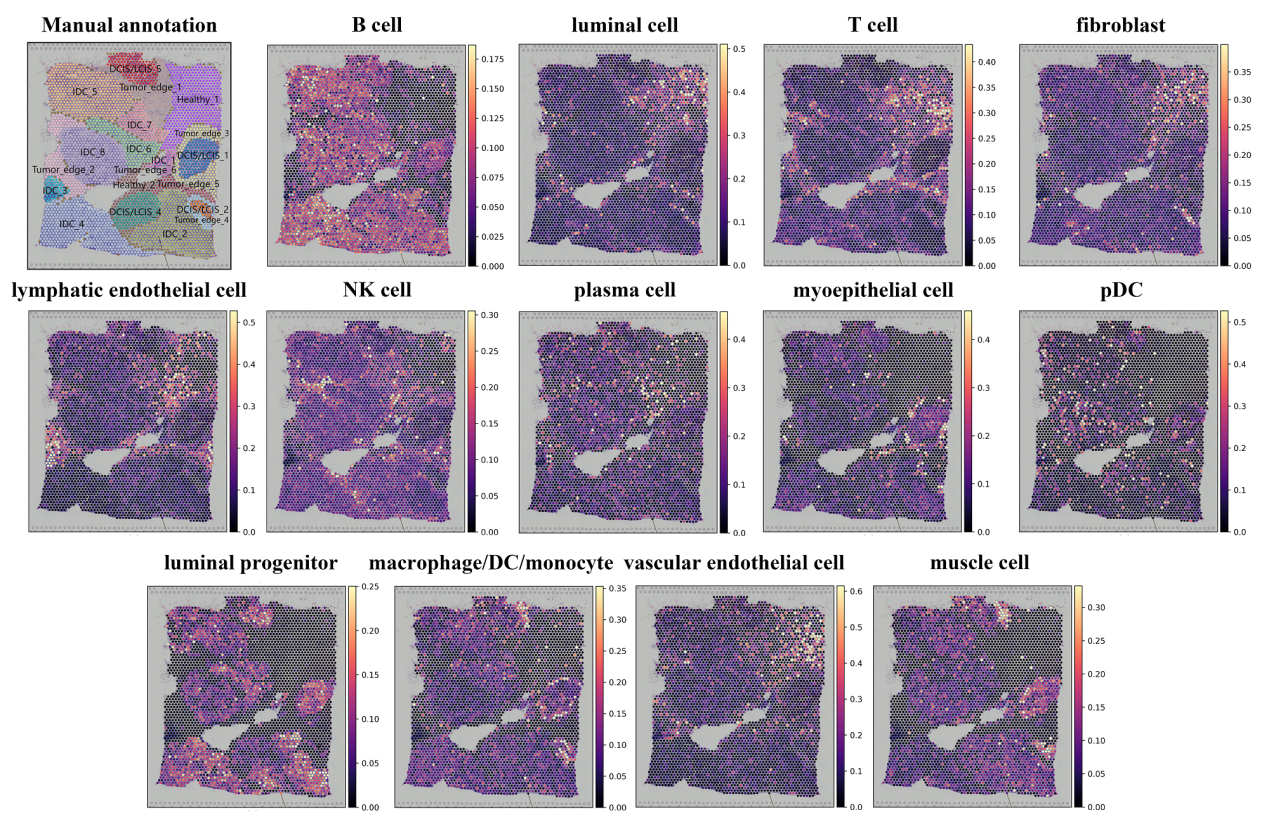
**

**Fig S12.** Manual annotation and spatial distribution of major cell types mapped by Tangram.

**Supplementary Table**

| **Datasets** | **Region** | **Cells / Spots number** | **Gene number** | **Cell type number** | **Cells number from scRNA-seq** |
| --- | --- | --- | --- | --- | --- |
| MERFISH | mouse brain medial preoptic area | 4,504 (1,107,256) | 135 | 6 | 1,691 |
|  |  | 4,676 (1,102,253) |  |  |  |
|  |  | 5,066 (1,118,256) |  |  |  |
|  |  | 5,199 (1,132,256) |  |  |  |
|  |  | 4,836 (1,132,256) |  |  |  |
|  |  | 4,743 (1,131,256) |  |  |  |
|  |  | 4,495 (1,099,255) |  |  |  |
|  |  | 4,455 (1,104,255) |  |  |  |
|  |  | 4,666 (1,101,256) |  |  |  |
|  |  | 4,290 (1,111,256) |  |  |  |
|  |  | 4,349 (1,126,256) |  |  |  |
|  |  | 4,275 (1,112,256) |  |  |  |
| seqFISH+ | Cortex of mouse brain | 523 | 10,000/6,000/3,000 | 6 | 1,691 |
| DLPFC(151673) | Human dorsolateral prefrontal cortex (DLPFC) | 3,639 | 33,538 | 33 | 78,886 |
| Mouse Brain Anterior and Posterior | Mouse Brain | 2,695 / 3,355 | 32,285 | 40 | 116,921 |
| Human Breast Cancer | Human Breast | 3,798 | 36,601 | 13 | 45,647 |

**Table S1.** The general summary of the spatial transcriptomics datasets we used in the experiments.

**Table S2.** The ablation study on seqFISH+ and MERFISH

| Methods | seqFISH+ | | | | MERFISH | | |
| --- | --- | --- | --- | --- | --- | --- | --- |
|  | 10000 genes | 6000 genes | 3000 genes | 100 μm | | 50 μm | 20 μm |
| STCGAN | 0.150 | 0.162 | 0.197 | 0.146 | | 0.175 | 0.240 |
| STCGAN-w/o-*L_cyc_* | 0.220 | 0.197 | 0.202 | 0.236 | | 0.276 | 0.368 |
| STCGAN-w/o-*L_d_* | 0.158 | 0.170 | 0.203 | 0.152 | | 0.181 | 0.248 |
| STCGAN-w/o-*L_reg_* | 0.165 | 0.182 | 0.205 | 0.156 | | 0.184 | 0.254 |

*Note*: The experiments were conducted on seqFISH+ and MERFISH, measured by RMSE score.
